# Impacts of gender, weather, and workplace differences in farm worker’s gear

**DOI:** 10.1186/s40101-015-0074-2

**Published:** 2015-11-09

**Authors:** JuYoun Kwon, Hee Sok Park, Sun-Hwa Kim, Kyung-Suk Lee

**Affiliations:** Ulsan National Institute of Science and Technology, School of Design and Human Engineering, Ulsan, , South Korea; Department of Industrial Engineering, Hongik University, Seoul, South Korea; Department of Fashion Design, Sunchon National University, Suncheon, South Korea; Department of Agricultural Engineering, National Institute of Agricultural Science, Jeollabuk-do, South Korea

**Keywords:** Elderly workers, Agricultural accidents, Work environments, Clothing, Weather, Greenhouse, Gear, WBGT

## Abstract

**Background:**

The farmers cannot help working in outdoor conditions which have high humidity and solar radiation during the harvest period. Wearable items including clothing are the nearest environment of human body, and to understand the current state of them can be a way to set up an active prevention strategy against the health risk from heat stress in summertime agriculture. The aim of this study was to investigate the work wear and accessories which the elderly farmers used during agricultural working.

**Methods:**

One hundred twenty farmers (49 males and 71 females) working in nine separate sites on different days took part in this study. The average age of subjects was 61 years old. We examined the types of working posture, clothing, and items that the farmers used and/or wore. We also interviewed the farmers to know why they used such items while working.

**Results:**

The results of this study were as follows: (1) Farmers worked in the thermal environment which was over wet bulb globe temperature (WBGT) reference value, and the farmers could suffer heat stress due to workload induced from wearing conventional long-sleeved shirts and long trousers which were 0.66 clo in average under this summertime working thermal condition. (2) The farmers tended to change the layer of upper clothing for adapting to weather condition. (3) The types of footwear used seemed to be related with facilities as well as weather, and farmers tended to wear lighter footwear when the weather is hotter or when they work in PVC greenhouse. The majority of elderly farmers wore loafers and rubber shoes which had indistinguishable thin soles. (4) The types of hats showed the difference between facilities as well as gender and only 31.7 % of all participants used long brims. (5) Korean elderly farmers did not use any active cooling item as agricultural auxiliary tools in summer harvesting time.

**Conclusions:**

Korean elderly farmers worked in poor surroundings which could threaten their health and safety and seemed not to adjust their workload and clothing during summer harvest season. Thus, it would be necessary to monitor individual responses in order to ensure that the risk of heat stress is prevented.

**Electronic supplementary material:**

The online version of this article (doi:10.1186/s40101-015-0074-2) contains supplementary material, which is available to authorized users.

## Background

Agriculture is classified as a hazardous industry worldwide according to the National Institute for Occupational Safety and Health (NIOSH) [1] and ILO [2]. However, the farmers are vulnerable compared with people working in mining and construction industries which are the main fields focused on safety and health [1–4], and the rates of fatality in agriculture, mining, and construction in 2004 were 30.1, 28.1, and 11.9 per 100,000, respectively [1]. The farmers are exposed to extreme weather conditions, machine accidents resulted from farm mechanization, and so on, while they experience plenty of health problems [5]. Manual workers are still required for harvesting and mowing of vegetables and fruits in a lot of agricultural regions [6]. The farmers usually work in outdoor conditions which have high humidity and solar radiation, and thus, the body heat can be produced and preserved at that time depending on the work clothing which the farmers put on [7]. Furthermore, climate change, especially heat rather than cold, has induced the problem on health [8–11].

One of the hazardous factors would be the injury mortality related to extreme environmental conditions. The mean counts of daily mortality were 215 persons in South Korea from 2000 to 2007 [12] and 740 persons in France, for 20 days of August 2003 [13]. Generally, heat-related disorders occur in summer, and they often affect the workers working in the construction site and the fields. Especially, the agricultural work should be conducted by periods of ripening, and the work is carried out for long hours even in extreme weather such as hot and humid conditions. Many severe disorders and deaths occur in the farming industry due to the nature of agricultural working environments [4, 14–16]. Sixty-eight farmers died due to illness related to heat in the USA from 1992 to 2006 [17]. This situation is not different in South Korea. The number of patients with heat-related illnesses during the summer of 2012 was 984 people [18].

If excessive heat from physical activity or ambient environment is obtained by the human body, clothing should be adjusted to promote heat loss through behavioral approach. Heat stress is considered heat load. Heat stress can occur when human body temperature increases through six fundamental parameters such as activities, air temperature, humidity, air velocity, radiant temperature, and clothing [9, 19, 20]. If cooling down is not enough through perspiration and convection, it can cause discomfort and produce thermal strain by which death is able to be induced. Especially, while they are exposed to hot working environment, physical working activity and mental capability can be reduced, and thus, the risk of accident and heat-related illnesses can rise. Many studies show that physiological responses of elderly people are weaker than young people during working in the hot environmental conditions [21–23]. The heat would be fatal to elderly population working in the Korean agricultural environment which consists of the manual work.

Therefore, safety and health in a hot environment should be assured to the elderly farmers, and it is important to assess this environment. Mitigation of heat stress in the farming industry would be possible if the problems of the current thermal environments to which farmers are exposed to are elucidated. Wearable items including clothing are the nearest environment of the human body, and to understand the current state of them can be a way to set up an active prevention strategy against the health risk from heat stress in summertime agriculture. The health risk from heat stress in summertime agriculture is analyzed through the detailed investigation on environmental conditions, clothing such as upper and lower clothing, footwear, hat, used accessories, and work postures which can be used for estimating workload. The aim of this study was therefore to investigate the work wear and accessories which the elderly farmers used during agricultural working. It was hypothesized that elderly farmers would choose their clothing depending on gender, weather, and facilities. It would help in improving working environment for elderly farmers and provide the practical information to the manufacturer of clothes and other accessories for agriculture workers.

## Methods

### Participants

One hundred twenty farmers (49 males and 71 females) working in nine separate sites on different days took part in this study. The percentages of less than 50 years old, 50s, 60s, 70s, and more than 80 years old were 13.3, 23.3, 31.7, 29.2, and 2.5 %, respectively. The average age of the subjects was 61 ± 11.6 years old. The number of workers and the proportion of the crops and the facilities are shown in Table 1. Nine surveys were conducted in the six regions of South Korea from July 2012 to September 2012.

**Table 1:** Frequency and rate of participants, and age by facility, gender, and crops

		Frequency, person	Proportion, %	Age, years old (SD)
Gender	Male	49	40.8	61 (10.9)
	Female	71	59.2	62 (12.1)
Facility	Field	56	46.7	65 (10.7)
	PVC greenhouse	64	53.3	59 (11.9)
Crops	Chili	27	22.5	60 (11.8)
	Lettuce	6	5.0	62 (15.9)
	Apple	9	7.5	64 (8.9)
	Oriental melon	46	38.3	57 (11.0)
	Weed scraping	10	8.3	68 (8.6)
	Flower	6	5.0	73 (3.5)
	Others^a^	16	13.3	69 (8.9)

^a^Others includes black bean, tomato, bracken, grain sorghum, sweet potato, green pumpkin, and rice

### Study design and survey procedure

The types of crops were chili, lettuce, apple, oriental melon, mowing, flower, and so on (Table 1). The target of this study was the elderly farmers who worked in PVC greenhouses and fields on site.

Environmental conditions were measured at a height of 1.2 m above the ground in each site. Environmental measurements for surveys 1, 2, 4, 5, 6, and 7 were conducted in PVC greenhouses, and the measurements for surveys 3, 8, and 9 were conducted in the fields. Environmental measurements were conducted throughout work patterns. For example, the environmental conditions for some surveys were measured for several hours during early morning. Dry (*T*_a_) and wet bulb (*T*_nwb_) temperatures and globe temperature (*T*_g_) were measured by INNOVA MM0030 transducer. Wind speed (*v*_a_) was measured by using an anemometer (INNOVA MM0038 transducer). Wet bulb globe temperature (WBGT) was calculated by using Eq. 1 [20].

1 $$ 0.7 \times {T}_{\mathrm{nwb}} + 0.2 \times {T}_{\mathrm{g}} + 0.1 \times {T}_{\mathrm{a}} $$

First, we contacted the head of each village and asked the working hours and days (namely a busy period) of the majority of the village’s farmers before visiting each village because the farm work is usually decided depending on the periods of ripening. When we arrived in a region, environmental measurement equipment was set up in the working environment (e.g., a field or PVC greenhouse). We walked or drove around the region to find the farmers who worked in these environments, and the distances between farmers and an environmental measurement site varied approximately from 2 to 3000 m. The farmers’ consent for the study was asked and a less than 5-min interview about their clothing information was conducted. We examined the types of working posture, and the types and the number of clothing and items. We also interviewed the farmers to know why they used such items and each survey took about 5 min per person.

### Estimation of workload and clothing insulation

The types of crops and subjects’ working postures were observed, and metabolic rate (activity) was estimated using ISO 8996 [24]. The metabolic rate for picking fruits or vegetable and weeding was 165 W m^−2^ which was used as the baseline metabolic rate. The metabolic rate for each working posture was as follows: 0 W m^−2^ for sitting, 10 W m^−2^ for kneeling, 10 W m^−2^ for crouching, 15 W m^−2^ for standing, and 20 W m^−2^ for standing stooped according to ISO 8996 [24]. Then, these metabolic rates for the working postures were added. For example, the metabolic rate for “stand stooped” was the baseline one + additional one (e.g., 165 W m^−2^ + 20 W m^−2^ = 185 W m^−2^). The metabolic rates for lotus position and standing while bending the waist were not in the ISO 8996, and thus, the metabolic rate of lotus position was considered to be the same as that of sitting, and the metabolic rate of standing while bending the waist was considered to be the same as that of standing stooped.

“Lotus position” was classified when the farmers sat on the ground during their work. “Sitting” was classified when their legs’ angle was less than 90 ° with a thick foam seat pad or a chair. “Squatting” was classified when their legs’ angle was less than 90 ° without any thick foam seat pad or chair. “Standing” was classified when their legs’ angle was 180 ° and their body angle was nearly 180 °. “Standing stooped” was classified when their legs’ angle was between 90 ° and 180 °. “Standing while bending the waist” was classified when their legs’ angle was 180 ° but their body angle was nearly 90 °.

The questionnaire for the interview consisted of the types and number of upper clothing and lower clothing, lengths, and the types of hat, footwear, and accessories (Additional file 1). It was investigated while the farmers worked in the fields or PVC greenhouses. Clothing insulation (clothing; clo) was estimated based on the results of the questionnaire [25]. Clothing insulation was calculated after the clothing insulation for each clothes, and the accessories were found and added them up altogether.

### Statistics

Mean values, standardization values, and frequency were analyzed for age, gender, and facility. Gender, facility, and wearing a hat were performed as the dichotomous variables. Age, working posture, and footwear were performed as the ordinal variables. Numbers from 2 to 7 were given for sitting, kneeling, squatting, standing, standing stooped, and standing while bending the waist, and numbers from 1 to 6 were given for slipper, rubber shoes, loafer, running shoes, boots, and others. The correlation of Kendall’s *τ*_c_ was used for the ordinal variables, and Spearman’s correlation analysis was used to determine the measure of association among gender, age, WBGT, clothing insulation, facility, footwear, the layer of upper and lower clothing, and wearing hats using a SPSS package.

## Results and discussion

### Environmental conditions and working posture

The environmental conditions for nine cases are shown in Table 2, and the weather data from Met Office in each region were also shown for the supportive data. In the case of the measurement at 1.2 m above the ground, the range of air temperature (*t*_a_) was from 23.4 to 32.2 °C and relative humidity (ø) was from 52.9 to 87 %. The range of mean radiant temperature (*t*_r_) was from 25.5 to 42.8 °C and wind speed (*v*_a_) was from 0.11 to 0.95 m s^−1^. WBGTs were from 20.6 to 30 °C. The values of four cases were higher than the WBGT reference value, inferring that the farmers worked in the thermal environment that could make them suffer heat stress (Table 2). The percentage of heat-related illness occurred approximately 74 % in a study for considering loss cost induced by occupational disorders [3]. Environmental conditions for surveys 1 to 9 varied and surveys 2, 4, 5, and 6 had WBGTs above the reference of ISO 7243 [26]. Depending on the WBGT value, the ratio of the work to the rest should be different below 38 °C for maintaining the internal body temperature. The allowed time for heavy work was less than 1 h, and this time decreased by 4 or 5 min as WBGT value increased 1 °C [9, 27]. Furthermore, the farmers feel the greatest discomfort when working under the sunlight (e.g., 92 %) and high temperature (e.g., 83 %) [15]. Based on the previous studies, one can conclude that surveys 1 to 6 had the WBGT values over 25 °C, and conducting agricultural work in these environmental conditions has negative influence on efficiency and productivity as well as health and safety of the farmers. The excessive thermal strain by the farmers is predicted, and it would reach to the threshold limit value. Parsons [28, 29] recommended the assessment of heat stress through ISO standards. Further analysis is required using ISO 7933, [30] and one needs to monitor individual physiological responses through ISO 9886 [31] if WBGT value is over reference values of ISO 7243 [26]. Furthermore, oxygen consumption tends to go up as air temperature increased [32]. Whereas energy expenditure such as resting energy expenditure and activity metabolic rate lessens with aging [33]. Therefore, safety and health in hot environment should be assured to elderly farmers. The current study was conducted in order to grasp the exact state of summertime working environment in Korean rural areas so that one can recognize that the outdoor working environment can endanger the elderly farmers. For an in-depth assessment to ensure the health and safety of the farm workers in the environmental condition, one would monitor individual physiological responses as a requirement.

**Table 2:** Mean environmental measurements

Survey	Weather station of Met Office			Measurements at 1.2 m above the ground				WBGT, °C
	*T*_a_, °C	Ø, %	*V*_a_, ms^−1^	*t*_a_, °C	ø, %	*t*_r_, °C	*v*, ms^−1^	
1	25.6 (1.54)	81.7 (7.96)	1.98 (1.000)	26.9 (0.45)	87.0 (3.01)	27.5 (0.57)	0.11 (0.069)	25.8 (0.39)
2	30.9 (0.68)	63.2 (3.66)	3.17 (0.698)	32.2 (1.39)	64.7 (5.10)	42.8 (5.14)	0.75 (0.268)	29.8 (1.27)
3	28.8 (0.75)	66.5 (1.52)	5.77 (0.314)	28.7 (0.68)	73.8 (1.70)	31.6 (2.21)	0.61 (0.272)	26.5 (0.70)
4	–	–	–	30.9 (2.11)	80.0 (5.41)	32.0 (3.60)	0.19 (0.079)	29.0 (1.66)
5	–	–	–	29.3 (2.79)	81.5 (18.29)	30.4 (3.43)	0.11 (0.035)	28.5 (2.09)
6	–	–	–	31.8 (0.90)	78.6 (2.58)	34.6 (2.11)	0.12 (0.037)	30.0 (0.83)
7	–	–	–	24.0 (1.72)	86.4 (7.32)	25.5 (3.05)	0.22 (0.141)	23.0 (0.71)
8	27.4 (1.01)	54.8 (6.97)	2.72 (0.818)	23.4 (1.14)	65.8 (5.20)	26.5 (5.44)	0.95 (0.412)	20.6 (1.39)
9	20.4 (3.69)	40.3 (4.76)	1.70 (0.390)	23.9 (1.98)	52.9 (7.85)	42.5 (4.44)	0.74 (0.313)	22.1 (1.31)

All values shown in brackets mean SD. Environmental conditions of surveys 3, 8, and 9 were measured in fields but others were recorded in PVC greenhouse

*Survey 1* harvest flower, measurement for 3 h from 14:45; *survey 2* harvest of lettuce, measurement for 2 h from 15:45; *survey 3* harvest of various vegetables, measurement for 2 h from 06:40; *surveys 4*, *5*, and *6* harvest of oriental melon, measurements for 2 h from 16:45, for 3.5 h from 06:00 to 08:30 and from 18:00 to 19:00, and for 1 h from 07:10; *survey 7* weed scraping, etc., measurement for 2 h from 16:45; *surveys 8* and *9* picking up apples, etc., measurements for 1 h from 17:20 and for 5 h from 08:00

Working posture of farmers consisted of sitting for 14 persons, squatting for 7 persons, standing for 26 persons, standing stooped for 26 persons, and standing while bending the waist for 47 persons, and the estimated metabolic rates were 165, 175, 180, 185, and 185 W m^−2^, respectively. The largest number of farm workers was shown in “standing while bending the waist.”

### Farmers’ working clothing

The types of clothing which the farmers wore were a bra, an underwear, a short-sleeved T-shirt, a long-sleeved T-shirt, a sleeveless T-shirt, a sportswear, a sleeveless vest, a raincoat, shorts, trousers, sportswear, and a raincoat for lower clothing. The average of clothing insulation of farmer’s clothes was 0.66 (0.143) clo (Table 3).

**Table 3:** The number of workers and clothing insulation by gender, facility and survey, and mean age by garments

			Upper clothing, person						Lower clothing, person				Clothing insulation, clo (SD)
			Underwear	Long-sleeved shirt	Sportswear	UL1^a^	UL2^b^	UL3^c^	Trousers	Sportswear	LL1^d^	LL2^e^	
Age (years old)	Mean		62	62	59	61	63	56	62	55	61	69	–
	SD		11.7	10.9	11.5	11.6	11.2	12.7	11.2	11.7	11.7	7.5	–
Gender	Male		16	24	4	28	20	1	33	13	45	4	0.63 (0.162)
	Female		49	45	2	15	46	10	57	9	65	6	0.68 (0.125)
Facility	Field		34	36	3	12	38	6	43	13	49	7	0.69 (0.167)
	PVC greenhouse		31	33	3	31	28	5	47	9	61	3	0.63 (0.112)
Survey	1		4	2	0	1	5	0	0	0	4	2	0.70 (0.104)
	2		5	5	0	4	5	0	8	0	9	0	0.74 (0.167)
	3		7	10	1	5	7	1	6	6	13	0	0.53 (0.227)
	4		7	4	2	3	7	0	8	2	10	0	0.70 (0.099)
	5		14	20	1	17	10	5	25	7	31	1	0.64 (0.131)
	6		1	2	0	4	1	0	5	0	5	0	0.63 (0.065)
	7		3	3	0	5	4	0	5	2	8	1	0.67 (0.094)
	8		5	3	1	1	6	1	8	0	7	1	0.59 (0.082
	9		19	20	1	3	21	4	25	5	23	5	0.63 (0.116)
	Total number		65	69	6	43	66	11	90	22	110	10	0.66 (0.143)

All workers wore brief, and it was not included in the layer of lower clothing

^a^UL1 means that workers wore one layer of upper clothing

^b^UL2 means that workers wore two layers of upper clothing

^c^UL3 means that workers wore three layers of upper clothing

^d^LL1 means that workers wore one layer of lower clothing

^e^LL2 means that workers wore two layers of lower clothing

The large number of farmers wore underwear and long-sleeved T-shirt in summer working environment, and there are 65 persons and 69 persons, respectively (Table 3). However, the number of farmers wearing sportswear was small in which there were only 6 persons. Female farmers wore underwear and long-sleeved T-shirts more than male farmers, but the former put on sportswear less than the latter. The average number of layers for upper clothing in each survey was from 1.2~2. The percentage of farmers wearing two layers was more than half, 66 persons.

The number of the farmers wearing trousers was 90 persons, preferring long trousers to shorts during their work (Table 3). However, the number of the farmers wearing sportswear was 22 persons, which was small. The average number of layers for lower clothing in each survey was from 1 to 1.3, and the percentage of the farmers wearing one layer was 87.6 % (Table 3).

### Farmers’ footwear and socks

The types of footwear which farmers wore were slippers, rubber shoes, loafers, running shoes, boots, and so on, and this order of footwear was related to the area of foot wrapped and thermal insulation. For example, the smallest area of foot was wrapped by slippers while boots wrapped the largest area of foot (Table 4). This footwear also had different thicknesses of soles. For example, rubber shoes and loafers had thin soles but running shoes and boots had thick soles. Loafers were used by the largest number of the farmers. The second largest number of farmers used rubber shoes and boots. Of the farmers, 77.5 % put on socks, and 85.9 % of females and 65.3 % of males put on socks (Table 4). Lee and Choi [34] studied the agriculture fatigue shoes and organized the footwear from the most frequently worn to the least frequently worn (e.g., rubber shoes, slippers, sneakers, and boots in order). It showed the conflicting results with the current study which organized it from the most frequently worn to the least frequently worn (e.g., loafers, rubber shoes/boots, and slippers in order). Both loafers and rubber shoes are the solid type which had indistinguishable thin soles between insole, midsole, and outer sole, and farmers of more than half out of all participants wore footwear without insoles which can be helpful to reduce fatigue [35]. Dunne et al. [36] pointed out that elderly people had a scant knowledge of the importance to have secure footwear, and Huebner et al. [35] showed that heel cushioning reduces muscle effort and delays muscle fatigue. Robbins et al. [37] and Goonetilleke [38] also pointed out that stability limits could be changed depending on the type and properties of footwear such as firmness and thickness of soles (i.e., they can have an influence on the stability of the pose).

**Table 4:** Mean age and proportions of workers wearing socks, footwear, hat, and accessories by gender and facility

		Age, years old		Gender, %		Facility, %		
		Mean	SD	Male	Female	Field	PVC greenhouse	Total, %
Socks		64	11.2	65.3	85.9	42.9	81.3	77.5
Footwear	Slippers	58	11.5	10.2	5.6	5.4	9.4	7.5
	Rubber shoes	61	13.2	18.4	29.6	12.5	35.9	25
	Loafers	63	11.8	20.4	45.1	37.5	32.8	35
	Running shoes	57	12.7	8.2	4.2	5.4	6.3	5.8
	Boots	62	9.6	38.8	15.5	37.5	14.1	25
	Others	69	4.9	4.1	0	1.8	1.6	1.7
Hat	No hat	61	13.2	40.8	38	25.0	51.6	39.2
	Baseball cap	66	8.4	32.7	5.6	21.4	12.5	16.7
	Bucket hat	62	8.4	10.2	5.6	12.5	3.1	7.5
	Sun cap	59	10.6	0	16.9	7.1	12.5	10
	Hat for farm work	61	13.1	4.1	25.4	26.8	7.8	16.7
	Towel	61	9.5	2.0	5.6	1.8	6.3	4.2
	Straw hat	63	10.0	8.2	2.8	5.4	4.7	5.0
	Others	61	0	2.0	0	0	1.6	0.8
Accessories	Belt	66	8.1	28.6	2.8	14.3	12.5	13.3
	Scarf/towel	69	5.7	6.1	11.3	10.7	7.8	9.2
	Arm sleeves	64	11.6	18.4	57.7	46.4	37.5	41.7
	Gloves	61	11.3	67.3	80.3	75.0	75.0	75
	Waist bag	72	4.4	2.0	5.6	1.8	6.3	4.2
	Mask	56	9.2	0	2.8	1.8	1.6	1.7
	Auxiliary tool	63	11.6	55.1	50.7	48.2	56.3	52.5

### Farmers’ hats and accessories

The types of hats which farmers wore were a baseball cap, a bucket hat, a sun cap, a hat for farmers, a towel, a straw hat, and others (Table 4). The percentage of the farmers wearing no hat during work was 39.2 %. The baseball caps were worn by a large number of male farmers, but the largest percentage of female farmers wore “hat for farm work.” More than 50 % of the farmers working in PVC greenhouses did not wear hats while 25.0 % of the farmers working in the fields did not wear hats. Accessories consisted of a belt, a scarf/towel, arm sleeves, gloves, a waist bag, a mask, and tools (Table 4). The kind of tools was a weed whacker, scissors, a hoe, a thick foam seat pad, picks, a rice-planting machine, an ice pack, a sickle, a shovel, a lumbar pad, and an umbrella integrated chair.

### Gender with gear

The layer of upper clothing showed a difference with gender (*p* < 0.05) and had a significant relationship with gender (rho = .388). Of the males, 57.1 and 2 % preferred to put on one layer and three layers of upper clothing, respectively, but 21.1 and 64.8 % of females wore one layer and two layers of upper clothing, respectively (*p* < 0.001). Socks on showed a difference depending on gender (*p* < 0.05). The different tendency was shown in socks on between female and male farmers (rho = .243). Footwear showed a difference with gender (*χ*^2^ = 15.858, df = 5, *p* = 0.007) and had a correlation with gender (*τ* = −.224). Female farmers wore lighter footwear than male farmers. The largest number of female wore loafers but the largest number of male wore boots. Choi et al. [39] studied labor burden of the farmers during harvesting chili in summer and showed that all female farmers wore rubber shoes. However, in the current study, the female farmers wore lighter footwear such as slippers, rubber shoes, and so on, which wrapped feet less, and the current study also showed that footwear had a significant correlation with gender.

Hat showed a difference depending on gender (*χ*^2^ = 33.720, df = 7, *p* = 0.000), but there was no relationship between them. Male farmers wore baseball caps while female farmers wore “hat for farm work” (e.g., 32.7 and 25.4 %, respectively). Diffey and Cheeseman [40] emphasized that it was important to use appropriate clothing and hats to avoid direct solar radiation and showed that the length of brim had different influences on the protection for the anatomical regions from the sun. In the current study, 39.2 % of the farmers did not use a hat, and wearing a hat showed a difference between genders.

### WBGT with facility and gear

Facility showed a difference between WBGT (*χ*^2^ = 113.750, df = 8, *p* = 0.000) and had a correlation with it (*τ* = −.934). PVC greenhouses had higher WBGT than fields. Clothing insulation showed a difference depending on WBGT (*χ*^2^ = 390.923, df = 344, *p* = 0.041) and had a correlation with WBGT (*τ* = −.191, Tables 2 and 3). Farmers tended to wear less clothing insulation as WBGT was higher. The number of layers for upper clothing showed a difference (*χ*^2^ = 27.880, df = 16, *p* = 0.033) and had a correlation with WBGT (*τ* = −.257). Footwear showed a difference depending on WBGT (*χ*^2^ = 67.726, df = 40, *p* = 0.004) and had a correlation with WBGT (*τ* = −.123). Farmers wore lighter footwear as the weather condition was hotter. The states of California and Washington provide the recommendation or guideline for being exposed to hot environment and selecting garments and personal protective equipment depending on air temperature. They take safety and health of the farmers seriously because being exposed to the hot weather can jeopardize the lives of the farmers [41, 42]. Several studies dealt with the importance of clothing in thermal environment exposure, and they suggested that there was a strong relationship between clothing insulation and environmental temperature such as WBGT value [20, 43]. The present study also showed the correlations between weather condition and clothing factors such as both clothing insulation and upper clothing rather than lower clothing. Elderly farmers seem to change their upper clothing for adapting to the change of weather. Kwon and Choi [44] showed the similar result about adjusting the upper clothing and lower clothing although the participants of 20s in a seated posture were exposed to the comfort range of environmental condition in a climatic chamber. Furthermore, a guideline from NIOSH [45] suggested that the farmers should not work in a place with high degrees of environmental temperature if they do not wear particular protective clothing with cooling function. The types of work uniform can increase or reduce the heat load because cardiac output increases by 30 to 70 % when body temperature goes up over 0.5 °C [46–49]. However, the Korean elderly farmer did not use any active cooling item during working although many functional cooling items such as cooling arm sleeve and cooling scarf are developed and popularly used for leisure activity. Especially, female farmers tended to wear more upper clothing than male farmers. Kjellstrom et al. [50] emphasized that the recommendation is related to the clothing which the farmers put on, and the farmers need to rest more if they wear more clothing. Accordingly, it was indicated that it is important to know how much clothing individuals wear.

Fleischer et al. [51] investigated the prevention practice on heat-related illnesses, and the rates of wearing long-sleeved upper clothing and long trousers were 19.5 and 92 %, respectively. However, the current study showed that the rate of wearing long-sleeved upper clothing was approximately 60 % (69 persons) and the rate of wearing long trousers was 75 % (90 persons). There were previous studies about elderly people’s physiological responses. Inoue et al. [52] showed that older men had lower sweat rate on their thigh compared with younger men, and the extremities of older men had decreased the capacity for sweat gland function. Although older people have decreased the capacity for sweat gland function on their extremities, high rectal temperature can occur to elder people due to the greater heat storage [23]. Long-sleeved upper clothing and long trousers generally have higher clothing insulation than short-sleeved ones. The period from June to August in summer of South Korea particularly is the busiest harvest time in rural areas and cannot help conducting the harvest with hand. High workload cannot also be avoided although the weather condition is extreme. Human body typically gets rid of the body heat through sweating and evaporation during heat stress, and thus, the farmers clothing, especially the long ones, plays an important role. CDCP [17] reported that the farmers often wear extra clothing, and thus, they are more exposed to the risks of heat-related illnesses. The average of clothing insulation in the present study was 0.66 clo which was slightly higher than the range of 0.4 clo to 0.7 clo from Choi et al. [39] because long-sleeved clothing and long trousers can provide not thermal but physical comfort through protecting the body from the sun, leaves, grass, insects, snakes, and so on. Wearing work uniform and keeping work posture with a heavy workload can aggravate heat load or alleviate the accumulation of heat stress depending on textiles and the composition of clothing. Therefore, it would be necessary to select functional fabrics such as a quick wicking and drying fabric which can provide low clothing insulation. Additionally, it was revealed that most working conditions that were investigated seemed unable to ensure health and safety of the elderly farmers due to natural weather conditions. So, it could be implied that potential fatal accidents related to the heat during agricultural work were not unexpected.

It is necessary to provide intervention after understanding their working environment. Therefore, functional work clothing which can reduce heat production on human body even during work should be developed and distributed to the farmers working in a hot environment. Also, it would be necessary to develop useful equipment like auxiliary cooling devices and devices for keeping less heavy work posture to mitigate health risk from hazardous working conditions. One can consider developing single complex equipment, which assorts cooling functions and work convenience.

### Facility with age and gear

Facility showed a difference between age (*χ*^2^ = 9.772, df = 4, *p* = 0.044) and had a correlation with age (*τ* = −.225). Older farmers tended to work in fields. Clothing insulation showed a difference with facility (*p* < 0.05) and had a relationship with facility (rho = .252). Clothing insulation (0.63 clo) in the PVC greenhouse was lower than that (0.69 clo) in fields (Table 3). The layer of upper clothing showed a difference in facility (*p* < 0.05) and had a relationship with facility (rho = .242). Although lots of farmers working both in the fields and in the PVC greenhouses wore two layers of upper clothing, the farmers working in the field seemed to prefer wearing two layers than those working in the PVC greenhouses. Footwear showed a difference with facility (*χ*^2^ = 15.117, df = 5, *p* = 0.010) and had a correlation with facility (*τ* = −.364). Lighter footwear was used more in the PVC greenhouses rather than in the fields. The large number of the farmers wore loafers or boots in the fields, but the largest number of the farmers in PVC greenhouses wore rubber shoes. Footwear had a significant correlation with facilities, and lighter footwear was used more in the PVC greenhouses rather than in the fields where 37.5 % of participants wore boots. It seemed that there was a difference in preference for the footwear depending on the workplace. Bailey and Hall [53] and TUC [54] noted that when workers conducted tasks for long periods of time, the workers can have trouble and agony with their lower extremities such as the feet, legs, pelvis, and lumbar due to footwear, which might be with matting for attenuating an effect on the feet. Korean elderly farmers in the present study seemed to wear footwear depending on weather and facility. So, the intervention would be required for the farmers to choose proper footwear to enlighten their work environment and tasks, and they would be informed that the type of footwear can have an influence on the fatigue of body.

Wearing a hat showed a difference with facility (*χ*^2^ = 8.844, df = 1, *p* = 0.003) and wearing hats had a relationship with facilities (rho = −.271), and hats were more used in the fields rather than in the PVC greenhouses (Table 4). Elderly farmers wore a hat with a shorter brim in the PVC greenhouse than in the fields. The type of hats showed a difference between facilities as well as gender. The type of hat was different between facilities, and the largest number of farmers wore baseball cap and sun cap in the PVC greenhouse (e.g., 12.5 and 12.5 %, respectively) and hat for farm work in the field (e.g., 26.8 %). Diffey and Cheeseman [40] suggested that the brim over 0.075 m could provide the protection for the nose and cheeks from the sun. However, only 31.7 % of all participants had long brims in the present study (i.e., sun cap (10 %) + straw hat (5 %) + “hat for farmers” (16.7 %) = 31.7 %). It is analogous to the result from the study of Fleischer et al. [51] in which 67 % of the farmers could hardly cover or never wore hats with a large brim. Previous studies showed that wearing hats have positive effects on human physiological responses, and it is necessary for the farmers to choose appropriate hats while they work during the day.

The limitation of this study was that in the case of clothing and work posture, the observation was done in a day for each person about 5 min. They could make the results various if the whole working hours were considered. This study was the user survey in a field, and the correlations of variables were low if compared with the measurement in a laboratory, and the power of statistical explanation for the relationships would be weak. In addition, workload was assumed for the quantification of work posture rather than the measurement of metabolic rate. Individual differences such as ages and body surface area were not also considered. For other supplementary information, long-sleeved upper clothing generally have higher clothing insulation than short-sleeved ones due to the body covered area by clothing which could be related to heat loss. The percentage of the body covered area by each clothing and the accessories tried to be estimated from ISO 9920 [25], but the percentage for the typical combination of clothing and accessories based on the result of the survey seemed to exceed 100 %. If the whole body covered area by a long-sleeved shirt (51 %), long trousers (44 %), a pair of loafer (7 %), gloves (5 %), and a hat (4 %) tried to be estimated, the percentage would be 111 %. It also happened to another combination of clothing and accessories such as a short-sleeved T-shirt (36 %), trousers (44 %), socks (14 %), arm sleeves (10 %), and gloves (5 %), and the percentage of whole body covered area would be 113 %. Due to them, body covered area which can affect heat loss of the skin could not be considered in this study.

## Conclusions

This study demonstrated how Korean elderly farmer’s clothing is influenced by weather, gender, and facility while the elderly farmers in Korean summertime are working under the risk of heat stress. Korean elderly farmers worked in poor surroundings which could threaten their health and safety and seemed not to adjust their workload and clothing during summer harvest season. It revealed the need to improve the working condition such as environmental condition, the working postures, and working gear. Thus, it would be necessary to monitor individual responses in order to ensure that the risk of heat stress is prevented.

The results of this study were as follows: (1) Farmers worked in the thermal environment which was over WBGT reference value, and the farmers could suffer heat stress due to workload induced from wearing conventional long-sleeved shirt and long trousers which was 0.66 clo in average under this summertime working thermal condition. (2) The farmers tended to change the layer of upper clothing for adapting to weather conditions. (3) The type of footwear used seemed to be related with facilities as well as weather, and farmers tended to wear lighter footwear when the weather is hotter or when they work in PVC greenhouse. The majority of elderly farmers wore loafers and rubber shoes which had indistinguishable thin soles. (4) The type of hats showed a difference between facilities as well as genders, and only 31.7 % of all participants used long brims. (5) Korean elderly farmers did not use active cooling item as agricultural auxiliary tools in summer harvesting time. Hence, the following recommendations would be supportive for preventing heat stress: (1) to wear clothing made with the high-tech fabric providing cooling function which can help in maintaining body temperature by absorbing and drying sweat quickly, (2) to use long brim hat for protecting farmers from the sun, and (3) to utilize auxiliary cooling items such as a cooling vest, etc. for maintaining an ordinary body temperature.

## Additional file

Additional file 1:
**Questionnaire for working clothing.** It is a survey questionnaire to collect the information of farmers' working posture, clothing, accessories and auxiliary tools. (DOCX 18.5 KB)
