# Supplementary material for: Impacts of gender, weather, and workplace differences in farm worker’s gear
Source: J Physiol Anthropol. 2015 Nov 9;34:39. doi: 10.1186/s40101-015-0074-2 (PMC4640324; doi:10.1186/s40101-015-0074-2)
Supplement: Additional file 1: — Questionnaire for working clothing. It is a survey questionnaire to collect the information of farmers' working posture, clothing, accessories and auxiliary tools. (DOCX 18.5 KB) [file 40101_2015_74_MOESM1_ESM.docx]

**Additional file 1. Questionnaire for working clothing**

**Date: Name: Gender**: Male/Female **Age**(Date of birth):

**Crops:**

**Working posture: (Sitting, Kneeling, Crouching, Standing, Standing stooped, Standing bending the waist)**

**Working Clothing**

|  | **Type of clothing** | **No. of layers** | **Types of necklines**  1)Crew neck  2)V-neck  3)with collar  4)Others( ) | **Sleeve/Slacks lengths** (Sleeveless/Short  /Right above the elbow/  Three-quarter/long) | **Way to do buttons**  1) no button  2) open a few button  3) open all buttons  4) fully buttoned | **Clothes fitness**  1) Tight  2) Loose  3) Baggy | **Tuck-in/out** |
| --- | --- | --- | --- | --- | --- | --- | --- |
| **Upper clothing** |  | First layer from the body |  |  |  |  |  |
|  |  | Second layer |  |  |  |  |  |
|  |  | Third layer |  |  |  |  |  |
|  |  | Fourth layer |  |  |  |  |  |
|  |  | Fifth layer |  |  |  |  |  |
| **Lower clothing** |  | First layer from the body |  |  |  |  |  |
|  |  | Second layer |  |  |  |  |  |
|  |  | Third layer |  |  |  |  |  |
|  |  | Fourth layer |  |  |  |  |  |
|  |  | Fifth layer |  |  |  |  |  |

| **Hat**.  (colour/no) | Yes / No  ( / ) | | | **Type of hat** | | Baseball cap/Bucket hat/Sun cap/Hat for farm work/Towel/Straw hat  Others: | | | |
| --- | --- | --- | --- | --- | --- | --- | --- | --- | --- |
| **Socks** | Yes / No | | |  |  |  |  |  |  |
| **Type of footwear** | Slippers/Rubber shoes/Loafers/Running shoes/Boots/Others | | | **Footwear length** | | ankle/middle of calf/knee/above knee/thigh | | | |
| **Belt** | Yes / No | | | **Auxiliary Tool** | | (No. of item: ) Yes /No | | | |
| **Accessories**  (No.) | Scarf/towel( ) | Arm sleeves( ) | Gloves( ) | Apron( ) | Waist bag( ) | | Mask( ) | Muff for calf( ) | Type of Auxiliary Tool( ) |
| **Material**(rubber/waterproof/cotton/blended fabric) |  | Left:  Right: | Left:  Right: |  |  | |  |  |  |
| Length/location and Size | Neck/Back | Below elbow/  Above elbow | On wrist/  above wrist | 10/ 20/ 30cm from knee |  | |  |  |  |
